# Behaviors of lunar regolith simulants under varying gravitational conditions

**DOI:** 10.1038/s41526-025-00501-z

**Published:** 2025-10-01

**Authors:** Ian P. Madden, Sathyashri Muruganandam, Amine Missaoui, Oliver Gries, Jonathan Kollmer, Olfa D’Angelo, Suman Sinha-Ray

**Affiliations:** 1 Low Gravity Sciences, Universities Space Research Association, 425 3rd Street SW, Washington DC, 20024 USA; 2 Glenn Research Center, NASA, 21000 Brookpark Rd, Cleveland, 44135 OH USA; 3 Department of Mechanical Engineering, The University of Texas at Austin, 110 Inner Campus Drive, Austin, 78712 TX USA; 4 Department of Physics, Universität Duisburg-Essen, Lotharstraße 1, Duisburg, 47057 Germany; 5 Erlangen-Nürnberg Institute of Multiscale Simulation, Friedrich-Alexander-Universität, Cauerstraße 3, Erlangen, 91058 Germany; 6 Institut Supérieur de l’Aéronautique et de l’Espace (ISAE-SUPAERO), Université de Toulouse, France, 10 avenue Edouard Belin, Toulouse, 31400 France

**Keywords:** Rheology, Glasses, Mechanical engineering, Aerospace engineering

## Abstract

Understanding the behavior of regolith in varying gravity conditions, is critical for space exploration and future missions. In this work, the gravity-driven hopper flow of lunar regolith simulant in different gravitational accelerations (terrestrial, lunar) is first observed experimentally. Numerical simulations (DEM) are then developed to understand the role which cohesive interparticle forces play in such gravity-driven flow, using the theoretical framework of granular Bond number. Qualitative comparison between a terrestrial experiment and numerical simulation validated this framework. Following that, we numerically studied the dynamic behavior under varying gravitational conditions (from terrestrial to lunar to asteroid gravitational accelerations). We find that this behavior is extremely sensitive to the interplay of the gravity conditions and the attractive/cohesive forces among particles. The numerical and experimental results show that the complex interaction of these forces can drastically change the dynamics of the material producing effects relevant for variable gravity applications.

## Introduction

All rocky celestial bodies began as clusters of regolith, which have continued to aggregate to vary degrees since their formation in the early solar system. Understanding the mechanical and rheological properties of this regolith is therefore an integral part of any mission’s success to these rocky bodies, whether it involves sample collection^1–3^, in situ resource utilization (ISRU)^4–11^, or the analysis of a planetary defense intervention^12–16^. Limitations of this understanding were particularly evident on September 24, 2023 when the spacecraft Origins, Spectral Interpretation, Resource Identification, Security, Regolith Explorer (OSIRIS-REx) returned to Earth to drop off a sample of asteroid regolith into the Utah desert, after a rendezvous in October, 2020 with the C-type asteroid Bennu^17^. The sample, collected within in the Touch-and-Go Sample Acquisition Mechanism (TAGSAM), weighs over 121.6 g and is the largest asteroid sample returned to Earth to date. However, the margins of error for the amount of sample collected using the TAGSAM varied greatly, between 60 g of material and up to 120 g^18^. Although this surplus is a welcomed surprise, it highlights the difficulty in designing mechanisms that must interact with granular material under non-terrestrial gravitational conditions, as the TAGSAM had passed a series of tests performed under the expected conditions on Bennu^19–21^. To decrease such margins of error, a more thorough understanding of the dynamic properties of granular materials, as found on asteroids, the Moon, and Mars, is needed.

Undoubtedly, the ubiquity of granular materials has ensured a very mature scientific field has developed here on earth concerned with its processing and transport properties. And considerable effort has already been devoted to understanding how various material properties effect the rheology of these systems. In the case of gravity driven flow through hoppers and silos, it has been determined that the mass flow rate for a generic granular material will obey a $\sqrt{g}$ rate law called the Beverloo law^22^, where *g* is the gravitational acceleration. Since the advent of Beverloo, there has been a significant amount of work performed on improving the description of gravity driven flows^23–40^. However, non-terrestrial, and specifically low-gravity driven flows, are often overlooked in these descriptions. Here we wish to point out one such aspect of low-gravity driven flows which Beverloo does not capture: that there may exist a critical gravitational acceleration below which no flow is observed due to jamming. And as previously alluded to, predicting when this onset of jamming and thereby clogging occurs is pertinent to the development of regolith handling procedures off-planet. Furthermore, our objective here is not to develop a closed form description of this gravity dependent flow rate, but to address a series of increasingly simplified hopper systems and describe the behavior through simple, universal, and general terms. Any plan to address improvements upon a Beverloo style mass-flow law will be covered in future works.

Among the methods often used to link the individual and bulk properties of granular media in gravity-driven flow, one of the most generally applicable is the granular Bond number, Bo_g_^41^, which compares the material’s cohesive interparticle forces against the materials own weight^42–44^. The versatility of this measure is that it makes no assumptions about the origin of these granular forces and therefore can be used to describe many possible dynamic rheological regimes, much like the fluid Bond number from which it is analogously derived. However, even this simple methodology can become complex in granular media as both of these forces are dependent on the granular media in question, varying with material properties like chemical composition and particle size leading to variation in inter-particle forces like cohesion. Furthermore, considering that most regolith or regolith simulant are heterogeneous mixtures of many different minerals, composed of particles of varying sizes, determining a unique Bo_g_ per granular material becomes a challenging problem. Thus, the potential for vastly different Bo_g_ and rheological responses to varying conditions arises with even small changes in properties^45,46^.

Here we design a hybrid experimental and simulation framework to perform this analysis, based on a typical quasi-two-dimensional (2D) hopper geometry, often featured in industry. Significant work on granular flow though this geometry has been performed^28–33^, including in various gravitational accelerations^34–40^; however, the majority of studies have focused on terrestrial or hyper-gravity scenarios, and the few that have focused on reduced gravity have viewed clogging and jamming as a result to be explicitly avoided^47^. Using this experiment, we test the dynamic behavior of a typical regolith simulant used in designing deep space missions, JSC-1A. We conduct tests under Earth gravity, but also under Lunar gravity using the partial-*g* active drop tower (GraviTower Bremen Pro (GTB)) available at the Center of Applied Space Technology and Microgravity (ZARM) (Bremen, Germany). We then design a matching discrete element methods (DEM) simulation model, in which we can easily vary gravitational acceleration. We observe strikingly different behaviors under different *g*-levels, confirming that cohesive particle interactions become dominant in reduced gravity.

## Results

### Drop tower experiment

Understanding a granular materials’ behavior, as it depends on gravitational acceleration, *g*, and particle properties, is paramount to working with regolith in low- (e.g., lunar) or micro-gravity environments—as will be the case in future deep space missions, notably including ISRU^4–7,9,10^. As a prototypical example of the problems a granular materials processing plant may face under lunar gravity conditions, we first present a quasi-2D hourglass experiment, performed on JSC-1A lunar simulant^48^, at gravitational accelerations of 1.0 G and 0.19 G (where G is the nominal Earth gravitational acceleration, G = 9.8 ms^−2^). We access 0.19 G, close to the gravitational acceleration on the Moon, using the ZARM GTB active drop tower^49,50^ (*cf*. Sec. 4.1 for experiment’s details).

**Fig. 1:**
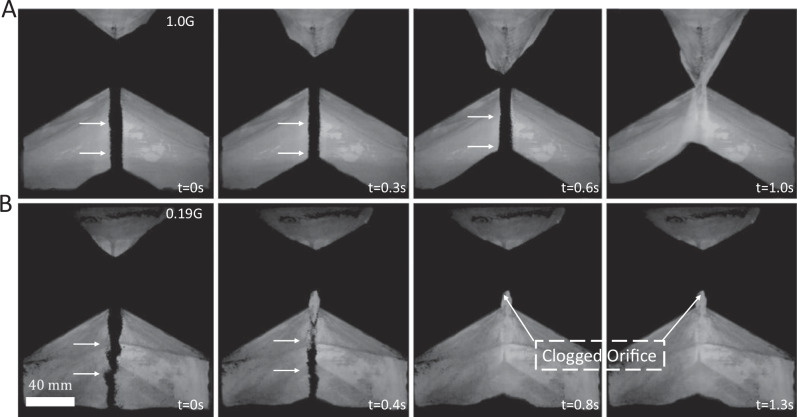
Hopper flow experiment for regolith simulant in Earth *vs*. Moon gravity. Photographs show JSC-1A lunar regolith simulant flowing through a macroscopic quasi-2D hourglass (hopper angle 60°) at (**A**) 1.0 G and (**B**) 0.19 G. The JSC-1A flows smoothly through the hopper-style hourglass to completion under nominal Earth gravity (see arrows in panels A, *t* = 0 *s*,0.3 *s*,0.6 *s*). Under lunar gravity, the simulant begins to flow smoothly (arrows in panels B, *t* = 0 *s*,0.4 *s*), but it then jams, clogging the orifice (arrows showing clogged orifice in panel B, *t* = 0.8 *s*,1.3 *s*). The system remains clogged until the experiment ends. The total duration of outflow in the Earth gravity experiment is approximately 0.9 s, and 1.3 s in the lunar gravity experiment. Lunar gravity conditions were performed using the ZARM GTB active drop tower (Bremen, Germany).

Under earth gravitational acceleration, the hourglass is flipped and simulant, initially in the top chamber, flows steadily through the orifice, sliding off the sloped hopper walls and through the throat unimpeded (see Fig. 1A, arrows pointing to smooth flow). The same experiment is then repeated under lunar gravitational acceleration (Fig. 1B). While the JSC-1A begins to flow continuously through the hourglass orifice (see arrows at *t* = 0 *s* and *t* = 0.4 *s*), the materials then clogs, forming an arch of jammed material just above the orifice (see arrows pointing to orifice at *t* = 0.8 *s* in Fig. 1B), arresting all subsequent flow. Some regolith simulant remains trapped in the upper chamber until the experiment is reset (see JSC-1A drop tower supplemental video).

As a lunar regolith simulant JSC-1A is known to have a high degree of shape and size polydispersity and is considered to be a fairly cohesive material^51^. Evidently, while it easily flows through hoppers under Earth gravity, JSC-1A is cohesive enough so that it might jam and clog the same hopper in reduced gravity. Simulants like JSC-1A are often used, here on Earth, to design regolith handling operations for deep space missions. These experiments conducted in lunar gravity clearly demonstrate that extrapolating results obtained on-ground using a typical regolith simulant will not hold true in lunar gravity. It is therefore of utmost importance to understand the fundamental interparticle forces which drive this difference in macroscopic behavior. To study how these microscopic forces are linked to bulk, process-scale phenomena, we use a lab-scale experimental setup of spherical particles to investigate particle-particle interactions which are then used to qualitatively validate a DEM simulation, and further provide insight into the flow characteristics of similar systems in variable gravity conditions.

### Micro-rheological experiment

To study the role interparticle interactions have on this clogging phenomenon (e.g., via interparticle cohesion, friction, and coefficient of restitution), we simultaneously conduct a series of DEM simulations (Numerical simulation) and, for validation of the simulations, a series of micro-rheological experiments in a quasi-2D hourglass (Micro-rheological experiment).

Because we are interested in measuring the limits of granular flow and in the interactions which controls these limits, to observe the dynamics on that grain-to-grain level requires a much smaller experimental setup than the macroscopic drop tower experiment, especially under 1.0 G conditions. Also, to reduce the computational complexity we study an alternative lunar simulant (Mars wind-drift) which is more uniform in composition and polydispersity than JSC-1A. In the following table we summarize the differences between the macroscopic experiment, microscopic experiment, and our simulation, for clarity (Table 1).

**Table 1:** Comparison of macroscopic experiment, microscopic experiment, and simulation hopper setups and granular media parameters

	Macro-experiment	Micro-experiment	Simulation
**Orifice diameter** *D*	10 mm	0.5 mm to 1.75 mm	0.5 mm to 1.75 mm
**Hopper angle** *α*	60°	75°	75°
**Granular media**	JSC-1A	Mars wind drift	—
**Particle distribution**	0.001 mm to 1.0 mm	0.005 mm to 0.5 mm	0.005 mm to 0.5 mm
**Median radius** *R*	0.05 mm	0.09 mm	0.09 mm
**Density** *ρ*	2900 kgm^−3^	400 kgm^−3^	400 kgm^−3^
**Gravity** *g*	1.0 − 0.19 G	1.0 G	1.0 − 0.01 G

**Fig. 2:**
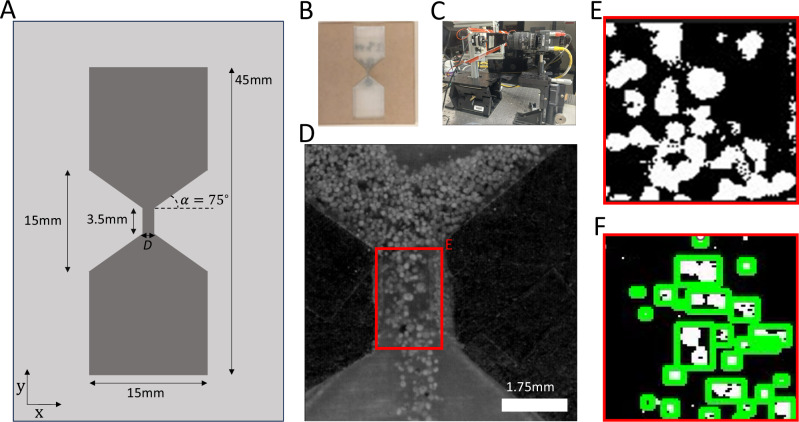
Micro-rheology experiment: hourglass and particle tracking pipeline. **A** Micro-hourglass dimensions: 45 mm tall and composed of two square chambers of width 15 mm, connected by a 3.5 mm channel of width *D*. The hopper opening angle *α* = 75°. The whole design is etched in acrylic to a depth of 0.75 mm. **B** Photograph of the micro-hourglass. **C** Imaging setup, using high-speed camera and macro lens. **D** Example image from high-speed camera of particles falling through the throat of the funnel. The particles’ velocity is measured for particles within the red outline. **E** Mask used as first step to identify particle locations within the red outline. **F** Particle segmentation used to track their locations and instantaneous velocity.

The hourglasses used for the micro-rheological experiments and validation of the simulations have a hopper angle *α* = 75°, and we vary the orifice diameter, *D* = 0.5 mm to 2 mm (Fig. 2A, B). The granular material used for the micro-rheological experiment is the Mars wind drift simulant (the reader is referred to its size chart and further characterization in^52^). We would like to iterate that the properties of JSC-1A and Mars wind drift are quite different. The Mars wind drift simulant was chosen for the micro-rheology experiments due its spherical shape, which allows a more direct comparison with DEM simulation.

The particle flows are imaged with a high-speed camera setup equipped with a macro-photo lens to resolve individual particles as they fall through the funnel throat (Fig. 2C, D). A sequence of machine learning and image analysis processes are applied to the raw images to detect and identify the position of each particle or particle clusters, as they fall through the throat region (Fig. 2E, F). Particle positions are tracked across frames to determine their velocity, and the velocities are averaged across the middle 50% duration of the observed flows to create flow profiles of particle velocity, *v*(*x*). Note that *v*(*x*) is measured in the negative *y*-direction (along the gravity vector) moving across the throat of the funnel in the positive *x*-direction. We record only the central 50% of the entire flow duration, discarding the initial and final 25% of the experiment as they feature inconsistent flow states (see Fig. S1 in supplementary information). The funnel diameter, $\bar{D}$, is non-dimensionalized by dividing it by the average particle diameter, $\bar{D}$ = D/〈d〉.

**Fig. 3:**
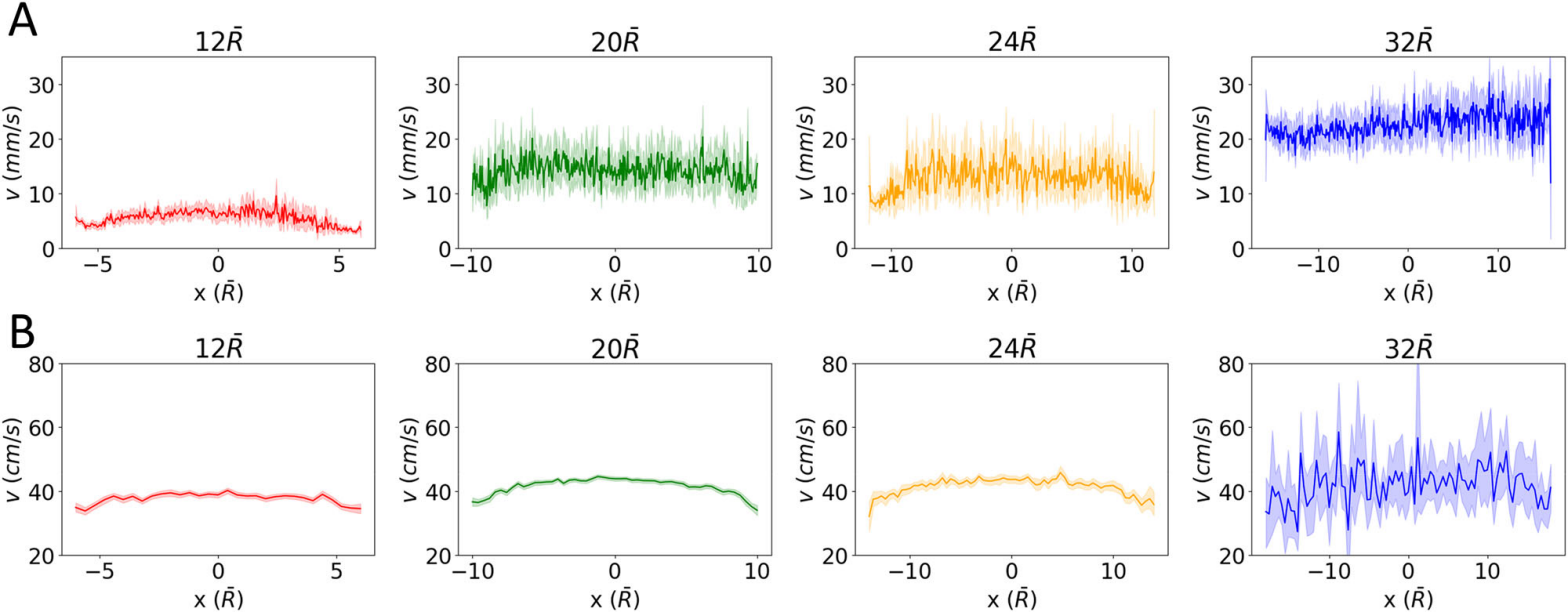
Particle flow profiles from micro-rheology experiment and simulation. **A** The flow profiles from experiment in the smallest funnel ($\bar{D}$ = 12, *D* = 0.75 mm) adopts a parabolic shape which proceeds to flatten out with increasing funnel size into the characteristic plug style profile expected of a flowing granular material at the largest size ($\bar{D}$ = 32, *D* = 1.75 mm). Shading represents a standard deviation calculated across 5 independent funnel flips. **B** Particle flow profiles from DEM simulation. Here, a cohesive interparticle force of *ϕ* = 0.001 Nm^−1^ is added to the force equation. Only for *ϕ* = 0.001 Nm^−1^, shown here, do we observe the parabolic to plug flow profile transition*:* if *ϕ* is too small, plug flow is observed regardless of funnel size*;* if *ϕ* is too large, the funnel clogs. Shading represents a standard deviation calculated across 16 independent simulations.

Flipping the hourglasses under earth gravity (1 G) conditions, we record the average flow profile for values of $\bar{D}$ ∈ [12,20,24,32]. We observe that for sufficiently large opening diameters, $\bar{D}$ > 32, the regolith simulant flows in a typical plug profile (Fig. 3), as expected for low-cohesion, spherical particles in this setup^43,44,53^. However, for $\bar{D}$ ≤ 32, the flow profile begins to transition away from plug flow and into a parabolic style flow profile^53,54^, Fig. 3A (the shading represents a standard deviation in the velocity profile). This characteristic transition from small opening diameters with parabolic flows to large opening diameters with flattened flows can be attributed to the interparticle cohesion and friction, and up to a certain extent, to the particles’ elasticity (coefficient of restitution)^53,54^.

It should be mentioned that many different mechanisms are at play and determining any single one as a dominant force behind the observed increase in effective cohesion would be difficult in this experiment. Therefore, we instead use this characteristic transition to qualitatively calibrate a simulation in which we can independently vary these interactions to identify the impact of each property on the flow characteristics and find which effect may eventually lead to the clogging as seen in (Fig. 1). It should also be mentioned that in this model, triboelectric charging caused by flow of grains past each other is not included. Note however that this effect would increase cohesion, eventually exacerbating clogging, we expect to include this effect in our future studies.

### Numerical simulation

The parameters needed for the simulation (particle friction, cohesion, coefficient of restitution) cannot be directly measured with high accuracy. In the case of the simulants used in this study, the difficulty is primarily due to a variety of different particle types. Note that these parameters are most often meant to qualitatively represent the macroscopic (bulk) behavior of a granular media, rather than the specifics of single particles as we are concerned with here. Therefore, we calibrate the DEM simulation based on the macroscopic results of our hourglass flow experiment.

**Fig. 4:**
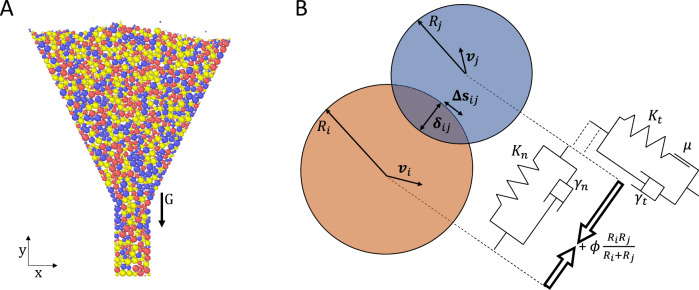
DEM simulation setup. **A** A funnel of throat diameter *D* = 1.75 mm and hopper angle *α* = 75 is filled with 1500 particles, ranging from *d* = 0.005 mm to 0.5 mm in diameter, and density *ρ* = 400 kg m^−3^. Gravity is applied in the negative *y*-direction. **B** The DMT model for contact forces between spheres of radii *R*_*i*_ and *R*_*j*_, with velocities **v**_*i*_ and **v**_*j*_, are split into normal and tangential components, governed by: their overlaps *δ*_*ij*_ and **∆s**_*ij*_; spring constants *K*_*n*_ and *K*_*t*_; damping coefficients *γ*_*n*_ and *γ*_*t*_; a frictional coefficient, *µ*; cohesion parameter, *ϕ*.

To calibrate these parameters, the setup used in the DEM simulation is a replica in-silico of the micro-rheology experiments (Fig. 4A). The interparticle force is computed based on the Derjaguin-Müller-Toporov (DMT)^55^ model (Fig. 4B), for it best approximates the interactions between hard (non-compliant) materials, akin to regolith simulants^56^ (see Figs. S2, S3 in supplementary information). This model is a modified Hertzian dash-pot potential, which includes an attractive force to account for the cohesive interactions between particles (e.g., Van der Waals). For two spheres of radii *R*_*i*_ and *R*_*j*_ with velocities **v**_*i*_ and **v**_*j*_, the forces are split into normal and tangential components governed by their overlaps *δ*_*ij*_ and **∆s**_*ij*_, Hertzian spring constants *K*_*n*_ and *K*_*t*_, velocity damping coefficients *γ*_*n*_ and *γ*_*t*_, a Coulomb frictional coefficient *µ*, and a cohesion parameter, *ϕ* (*cf*. Figure 4B). The form of this cohesion parameter is particularly important for our study, it is a pair-wise, inter-particle, attractive, contact potential, and thus it is distinct from the adhesive forces which act over large separations and are typically seen in wet granular materials^57^. The Hertzian elastic responses in the normal and tangential directions, *K*_*n*_ and *K*_*t*_, respectively, are determined via *K*_*n*_ = 4*E/*6(1−*ν*)(2 + *ν*) and *K*_*t*_ = 4*E/*4(1 + *ν*)(1−*ν*), where *E* is the elastic modulus of lunar regolith (60 MPa)^58^ and *ν* = 2*/*7 is the Poisson ratio. Similarly, *γ*_*n*_ and *γ*_*t*_, which control damping in the normal and tangential directions, are determined by the coefficient of restitution, *ϵ*, of the material. We vary the particles’ coefficient of restitution between *ϵ* = 0.1 and *ϵ* = 0.9. Likewise, we vary the Coulomb friction, controlled by the friction coefficient *µ*, between *µ* = 0.25 and *µ* = 0.90^59^. Finally, the cohesive force imposed through *ϕ* is varied along with the gravitational acceleration, *g*, to focus on the competitive effect of these two forces over the observed flow behavior. No other explicit attractive force (e.g., electrostatic) is included, such that *ϕ* assumes the singular role of cohesive control parameter in our simulation.

We first calibrate the model by repeating the experimental procedure in the simulation, extracting particles velocity from the DEM simulations, again like the experimental method for the micro-rheological experiment as described above, and average these velocities during the middle 50% of the overall flow duration within the throat region to create flow profiles. Our simulations are performed for varying funnel throat diameters, $\bar{D}\,$= 12−32 and with particles of matching density and polydispersity as the micro-rheological experiment. The hoppers are initially filled with 1500 poly-disperse spheres of radii *R* = 0.005 mm to 0.5 mm and of density *ρ* = 400 kg m^−3^. Here the particles radii are chosen to match the radii and average density of measured mars simulant. In reality, any type of simulant is a heterogeneous collection of materials of many different densities and sizes, and therefore, particles experience many different weight forces; in our simulation, only variation in size contribute to varying particle weight.

**Fig. 5:**
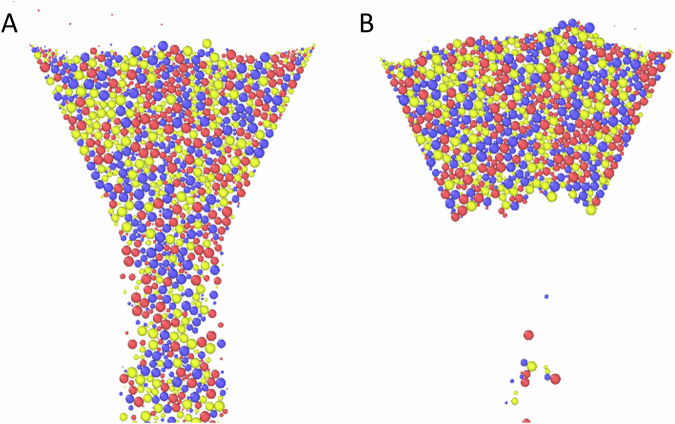
Effect of cohesive forces encoded in the *ϕ* parameter on the macroscopic hopper flow in our DEM simulation. **A** At low cohesion (*ϕ* < 0.001 Nm^−1^), the particles flow like a dry sand. **B** When *ϕ* is large *(ϕ* > 0.001 Nm^−1^), the grains are cohesive, and the hopper tends to clog.

In the simulation, the hoppers are initially plugged and randomly filled with the granular particles in an incremental fashion by inserting particles of random size and random initial position into the space above the funnel and then allowing gravity to settle them into the hopper until all 1500 particles have been placed. The plug is then removed, and flow profiles are recorded. The first goal of these numerical experiments is to calibrate our simulation parameters, specifically *ϕ* as the other parameters like, *ϵ*, and *µ* are determined by the material and have been found in previous studies^59,60^. We calibrate *ϕ* by qualitatively matching the flow behavior for varying opening diameters between the simulation and the micro-rheological experiment. We find that the phenomenon of the flow profile flattening as *D* increases, as observed in our micro-rheology experiment, only appears for *ϕ* = 0.001 N m^−1^ (Fig. 3B). For *ϕ* < 0.001 N m^−1^, the material flow is independent of opening diameter, so long as *D* is large enough to avoid clogging (Fig. 5A). On the other hand, for *ϕ* > 0.001 N m^−1^, the funnel immediately clogs (Fig. 5B). Using this simulation/experiment comparison as a macroscopic calibration procedure, we fix *ϕ* = 0.001 N m^−1^.

**Fig. 6:**
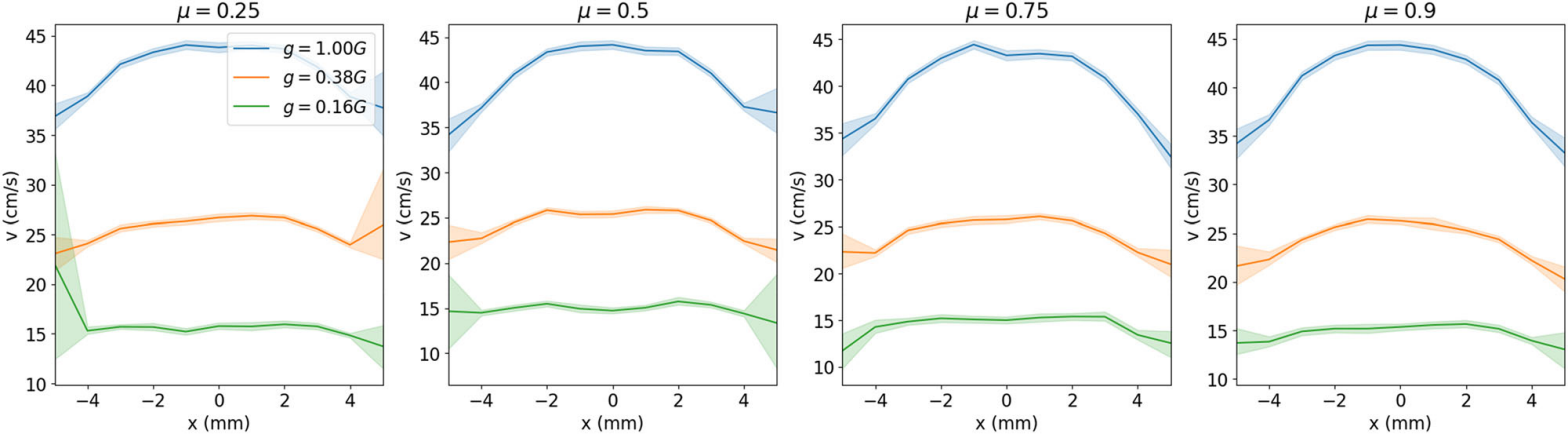
Flow velocity profile across hoppers at constant *ϵ.* The velocity profile *v*(*x*) of particles traveling through a *xy*-plane cutting across the throat of a funnel, *D* = 1.75 mm, at its mid-point along the *y*-direction, is recorded for *g* ∈ [1.0, 0.38, 0.16] G and *µ* ∈ [0.25, 0.5, 0.75, 0.9]. We fix *ϵ* = 0.5. At constant *µ*, the velocity decreases with *g*, as the driving weight force decreases. At fixed *g*, little variation in profile shape is observed across different *µ*, except that at low *µ* the particles along the wall tend to exhibit a large variation in velocity. Shading represents a standard deviation calculated for 16 repetitions.

Having determined the appropriate value of *ϕ*, we vary the other simulation parameters: *g*, *ϵ*, and *µ*^59,60^. We vary these parameters in sets of two and investigate the changes in macroscopic flow. The simulations are performed at a fixed funnel diameter, *D* = 1.75 mm, and again recording only the middle 50% of the flows. We first vary *µ* and *g*, holding *ϵ* constant at 0.5, and observe that for any fixed *µ*, the velocity profiles tend to flatten as *g* decreases (Fig. 6). This can be seen as a consequence of the reduction in shear force at lower gravity. Little variation is observed between profiles at fixed *g*, i.e., comparing curves of the same color among panels in Fig. 6. This indicates that the interparticle friction does not strongly modulate the flow profile. The same peak flow velocity is observed at 44 cm s^−1^, 26 cm s^−1^, and 15 cm s^−1^, for increasing *g*, regardless of *µ*. The only noteworthy change with *µ* is the increasing wall effect: at sufficiently low *µ*, the velocities vary more at the walls than near the center (see large shaded regions in Fig. 6 representing large standard deviation). We expected to observe a strong relationship between *µ* and flow velocity as has previously been recorded for gravity driven flows in both 2D and 3D geometries^61,62^; however, an important distinction separates our results from these other findings. Namely, we are concerned with modeling the micro-rheological system which is composed of a relatively small number of light weight particles, as opposed to a large volume of media discharging through a relatively small opening. As a result, the hopper does not contain enough volume of media to suitably establish how friction controls flow rate. None-the-less, friction strongly effects the flow during the initial period of the simulation, however the variation dissipates at the simulation proceeds, we attribute this to the very small amount of regolith used (see Fig. S4 in supplementary information).

**Fig. 7:**
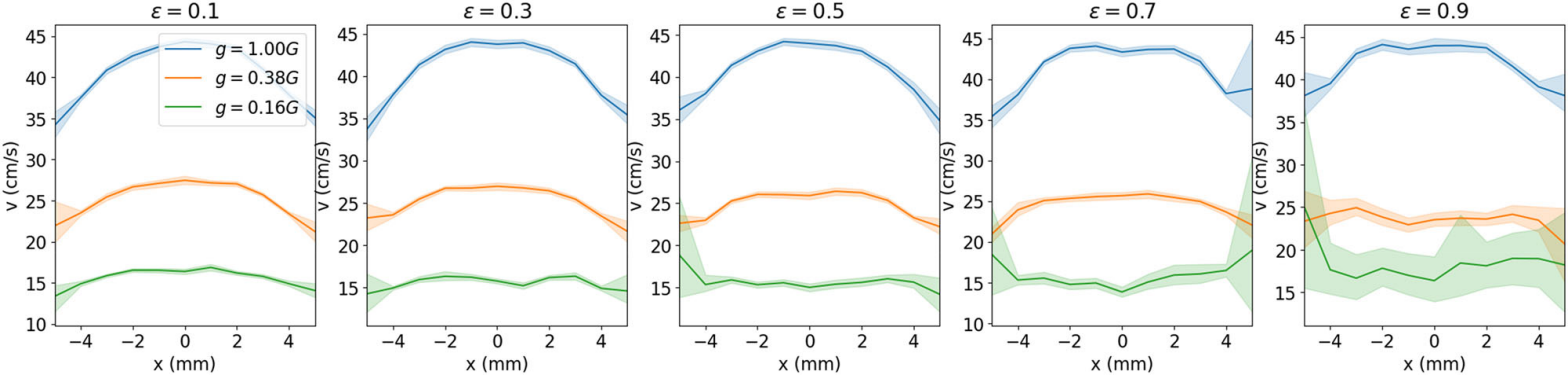
Flow velocity profile across funnel at constant *μ.* The velocity profile, *v*(*x*), of particles traveling through a *xy*-plane cutting across the throat of a funnel, *D* = 1.75 mm, at its mid-point along the *y*-direction, is recorded for *g* ∈ [1.0, 0.38, 0.16] G and *ϵ* ∈ [0.1, 0.3, 0.5, 0.7, 0.9], at *µ* = 0.5. As *ϵ* increases, the profiles flatten out. However, as *g* decreases, the profiles flatten out at earlier *ϵ*, such that at *g* = 0.16 G, the profile inverts from *ϵ* = 0.5. Shading represents a standard deviation calculated for 16 repetitions.

Next, we vary *ϵ* and *g*, holding *µ* constant at 0.5. We observe that under Earth gravity, the flow profile transitions from the classic parabolic style flow to a noticeably flattened profile as *ϵ* increases (Fig. 7).

Under martian gravity conditions (*g* = 0.38 G), the flow profiles are generally reduced due to the reduced accelerations, but phenomenologically, the same transition in profile is observed. However, the profile flattens out more rapidly, such that at the highest *ϵ* studied, the flow profile is nearly flat across the throat of the funnel, indicating that all particles are falling through the funnel at nearly the same speed. Finally, under lunar conditions, the velocities are the slowest, and the transition to a flat velocity profile occurs the earliest, around *ϵ* ≈ 0.3. Remarkably, from *ϵ* = 0.5 the velocity profile experiences an inversion to become an inverted parabola, with velocities slower at the center of the funnel and faster towards the walls. We attribute this change to two effects: (1) at such low gravitational acceleration, the profiles are already nearly flat at low *ϵ*; (2) high *ϵ* implies lower damping at each collision, so that it takes many interparticle collisions to dampen the same amount of energy. A larger number of collisions is more likely to occur in the center of the funnel than near the walls. Therefore, particles near the walls have larger variations in velocities, an effect seen for all curves at *ϵ* ≥ 0.5. Considering the relative effects of cohesion, friction, and restitution, we surmise that cohesion is the most influential force when we are concerned with the onset of jamming as it occurs between a small set of particles under low load.

## Discussion

We now focus on the effect that variation in the cohesion *ϕ* has on the macroscopic flow with the aid of the conceptual framework offered by the granular Bond number, Bo_g_^63–65^, defined as the ratio of cohesive force between a particle of radius *R*_*i*_ in contact with *N* particles of radius *R*_*j*_ over the gravitational force (weight) acting on particle *i* (of bulk density *ρ*):

1 $${{\rm{Bo}}}_{{\rm{g}}}=\left({\sum }_{j}^{N}3\phi \frac{{R}_{i}{R}_{j}}{{R}_{i}+{R}_{j}\,}\right)/\left(2\rho {R}_{i}^{3}g\right)$$

Immediately, a few complications with Bo_g_ become apparent within this definition. First, since the grains are polydisperse and both the attractive and gravitational forces vary with these particles (Bo_g_ depends on the particle properties of both particles *i* and *j*), there is not a unique Bo_g_ for our granular material. Second, because the attractive force is measured on a pairwise basis, it depends on the local configuration around the particle in question and is therefore a local measurement, whereas the gravitational force is globally invariant. Thus, a particle will always experience the same weight force, but it may feel vastly different attractive forces depending on the number and size of contacting particles. And similarly, the directions of these forces may also vary such that the net acceleration due to cohesive contacts do not always necessarily oppose the gravitational acceleration.

**Fig. 8:**
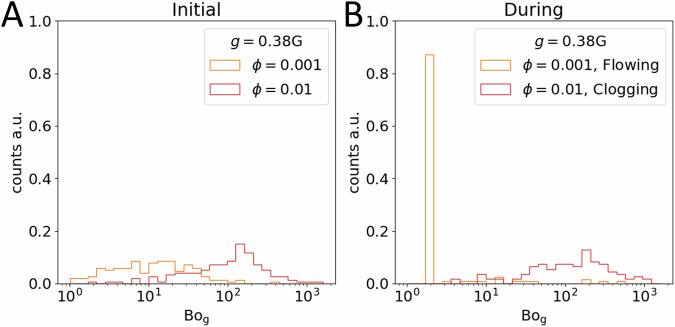
Distribution of granular Bond numbers. Defined as Bo_g_$={\sum }_{j}^{N}3\phi \frac{{R}_{i}{R}_{j}}{{R}_{i}+{R}_{j}\,}/\left(2\rho {R}_{i}^{3}g\right)$, we plot Bo_g_ between particles from the DEM simulations performed at *ϕ* = 0.001 and *ϕ* = 0.01, with *g* = 0.38 G. Distributions are gathered from both the initial simulation state before the hopper plug is removed (**A**), and during the simulation after the plug is removed and the grains may begin falling (**B**). **A** In the initial state both the *ϕ* = 0.001 distribution (orange curve, average Bo_g_ = 46.7), and *ϕ* = 0.01 distribution (red curve, average Bo_g_ = 451.9), span nearly three orders of magnitude and there is considerable overlap between them. **B** However, during the simulations performed at Martian gravity the less cohesive, *ϕ* = 0.001 distribution shifts significantly to lower Bo_g_ as grains flow smoothly through the hopper under free fall conditions making few if any contacts, average Bo_g_ = 27.4. In contrast the more cohesive and therefore more likely to clog *ϕ* = 0.01 distribution remains relatively unchanged, average Bo_g_ = 621.2.

Never-the-less, the Bond number can still be an instructive tool to predict behavior. We study the effect of the Bond number distribution for different gravity condition using different cohesiveness. For the sake of brevity, in this article, two different *ϕ*, 0.01 and 0.001 are shown for a Martian gravity condition. Figure 8 shows the distribution of granular Bond Number before and after removal of plug near the neck region. In the initial state both the *ϕ* = 0.001 distribution (orange) and the *ϕ* = 0.01 distribution (red) span more than two orders of magnitude but they also overlap considerably (Fig. 8A). The difference between the average Bo_g_ is also roughly the same as the difference between *ϕ*, in the *ϕ* = 0.001 case the average Bo_g_ = 46.7, whereas the average Bo_g_ = 451.9 for the *ϕ* = 0.01 case. However, because rearrangement may occur after the initial state—unless every particle is already jammed—there is an opportunity for these distributions to change. And indeed, switching to distributions gathered after the hopper seal is removed (Fig. 8B), we observe that in the less cohesive case (*ϕ* = 0.001) the distribution shifts significantly to lower bond numbers, average Bo_g_ = 27.4, as particles begin flowing and breaking their previously attractive contacts. In the more cohesive case the distribution does not change so drastically; instead, the average Bond number increases to Bo_g_ = 621.2.

**Fig. 9:**
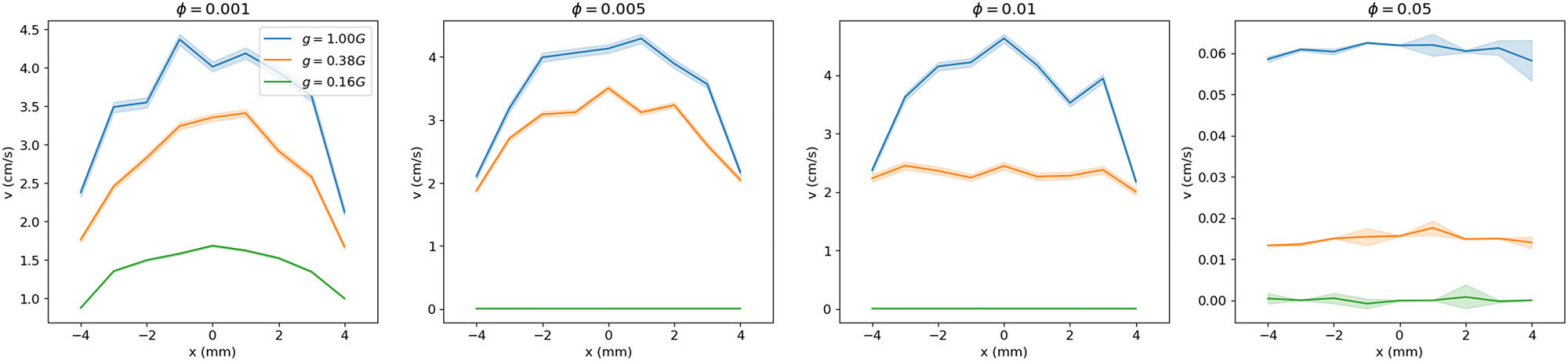
Flow velocity profile across funnel at varying *ϕ.* The velocity profile *v*(*x*) of particles traveling through a *xy*-plane cutting across the throat of a funnel, *D* = 1.75 mm, at its mid-point along the *y*-direction, is recorded for *g* ∈ [1.0, 0.38, 0.16] G and *ϕ* ∈ [0.001, 0.005, 0.01, 0.05], at *µ* = 0.5 and *ϵ* = 0.7. As *ϕ* increases, the profiles flatten out. As *g* decreases, the profiles also flatten out. Shading represents a standard deviation calculated for 16 repetitions.

Such variation in flow is obvious when investigating the velocity profiles under varying *g* and *ϕ*. We observe starting at low *ϕ*, the flow profiles are all parabolic, regardless of *g*; however, when the cohesive forces between particles increase, *ϕ* → 0.05 Nm^−1^, the particles become likely to jam above the orifice and clog the funnels, regardless of funnel diameter, *D*. Clogging abruptly stops or prevents flow, such that the flow profiles flatten out completely and the halting or viscous style flow develops. While Bo_g_ is a distribution due to the statistical nature of granular media, for gravity driven flow in variable gravity conditions, it appears that the probability to jam and the extent of the Bond number distribution are related. Specifically, weaker gravitational forces (green curves in Fig. 9) jam at *lower* cohesion, while strong gravitational forces (blue curves in Fig. 9) only jam at *higher* cohesion.

**Fig. 10:**
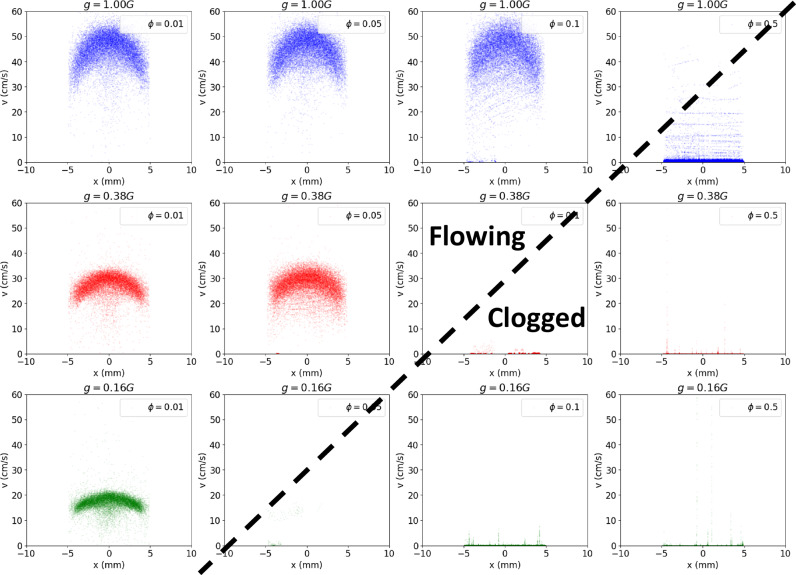
Clogging phase diagram. Particle velocities throughout funnel as a function of *ϕ* ∈ [0.01, 0.05, 0.1, 0.5] and *g* ∈ [0.16, 0.38, 1.0] G. As *ϕ* increases (left to right) and as *g* increases (bottom to top), the particles jam and arrest the flow, such that a clear clogging transition occurs as a diagonal line cutting across the (*ϕ,g*)-space.

When the funnel clogs, the motion of all the particles is arrested in the hopper. By tracking the velocity of all grains across the funnel, we propose a clogging phase diagram in the *g* and *ϕ* parameter space as Fig. 10. The average speed of all particles across the funnel is recorded for the entire duration of the simulation. Clogging appears for all accelerations, even 1.0 G, provided the material is sufficiently cohesive.

**Fig. 11:**
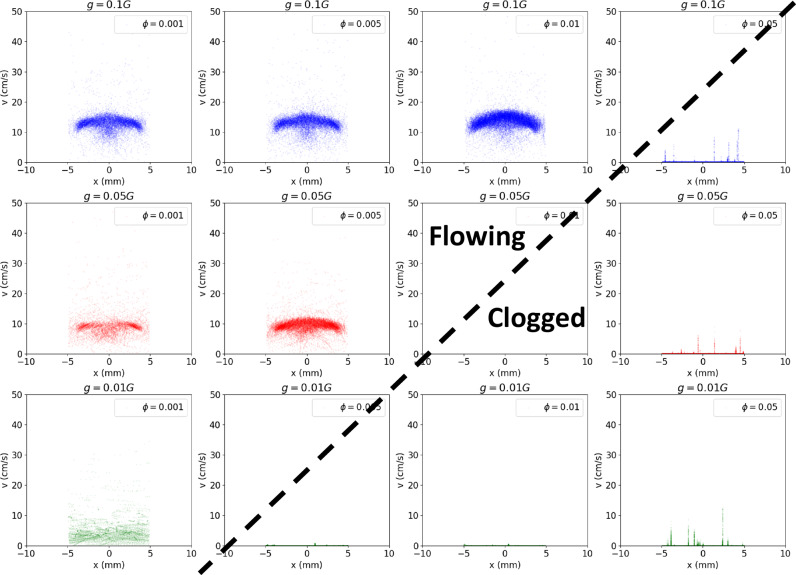
Clogging phase diagram at reduced gravity. Particle velocities throughout the funnel as a function of *ϕ* ∈ [0.001, 0.005, 0.01, 0.05] and *g* ∈ [0.01, 0.05, 0.1] G. As *ϕ* increases (left to right) and as *g* increases (bottom to top), the particles’ velocity exhibit the same behavior as above, such that a clear clogging transition occurs as a continuation of the diagonal line from the previous phase diagram, dividing the (*ϕ,g*)-space.

Going beyond lunar gravity, we extrapolate our findings to asteroid gravity, a condition critical for sample collection and planetary defense applications. A phase diagram where gravitational acceleration is decreased to 0.01G_0_ and at *ϕ* = 0.001−0.05 is studied (Fig. 11). In both figures, we observe a clear transition between flowing and clogging systems in the *g* and *ϕ* space, similar to the jamming transition phase diagram^66^.

At high *g* and low *ϕ* (upper left region in Fig. 11), a narrow band of velocities across the funnel develops as all particles are flowing through in a parabolic nature. Increasing *ϕ* (moving to the right in the diagram), the spread of velocities increases, showing higher variability between particles, until the hopper abruptly clogs at *ϕ* = 0.5, where only a few large positive velocities are recorded when single particles fall through intermittently. Decreasing *g* produces the same effect (going down in Figs. 10 and 11) with the onset of the clogging transition marked by the dashed line.

Observing a similar effect of increasing cohesion, *ϕ*, and decreasing gravitational acceleration, *g*, underlines the validity of the granular Bond number framework to interpret and extrapolate granular flows in reduced gravity. From the perspective of a scaling analysis this relationship makes intuitive sense as the the onset of jamming in the simplest of realizations requires interparticle cohesion to scale as the particle’s weight, (4*/*3)*πR*_*eff*_^3^ *ρg* ≈ 2*ϕπR*_*eff*_, implying *ϕ* ≈ [(2*/*3)*R*_*eff*_^2^ *ρ*]*g*. Here we have simplified the possible distribution in *R*’s to just one *R*_*eff*_, but the advantage of this simplification is that it shows how as gravity decreases, *ϕ* becomes more important. And conversely, for the present micro-rheological system, the *ϕ* required to initiate jamming and clogging must be larger as shown in Figs. 10 and 11. Also, this simple scaling rule shows that the transition between flowing to jamming in phi-g space is a uniform transition.

Granular bond number, Bo_g_, is a powerful tool often used to understand the jamming transition in granular media, and here we have shown its applicability towards understanding low gravity granular flows. The clogging observed in both experiment and simulation appears to be robust and may have considerable impact on future design and validation procedures for sample collection and return mission designs. In experiment two very different regolith simulants (JSC-1A and Mars wind drift) behave similarly when the funnel size and gravitational acceleration are appropriately tuned. Using an informed, but minimal, simulation model we then deduce that this is due to variation in inter-granular cohesive forces, captured by a single parameter *ϕ*. Thus, potential jamming can always occur provided the material is sufficiently cohesive or gravity is sufficiently weak, and this is without the inclusion of other explicit attractive inter-granular forces like electrostatics, which are expected to accentuate jamming and clogging. Expanding on these findings, work must be performed on the plethora of other regolith simulants, and other prototypical geometries must be studied. Of particular importance are the designs currently proposed under existing ISRU proposals for lunar missions which feature the complex processing of granular lunar regolith.

## Methods

### Macro-rheological experiment in lunar gravity

Macroscopic quasi-2D hourglass experiments are performed in 1.0 G and in low gravity, at 0.19 G ± 0.06 G. The low gravity experiments make use of the ZARM active drop tower, the GTB (Bremen, Germany). The hourglass itself has a height of 200 mm and a width of 180 mm and is composed of two chambers, both of the shape of angular quasi-2D hoppers, and is filled with approximately 60 g of JSC-1A lunar regolith simulant. The side walls of the hopper are made from aluminum and the front and back face are made of glass. The orifice diameter is *D* = 10 mm, and the space between the glass front and back panels is also 10 mm, such that the throat has a square crosssection. The upper chamber has an opening hopper angle of *α* = 60^◦^ and the lower chamber has an opening hopper angle of *α* = 120^◦^. Videos are captured at a resolution of 1024 × 768 pixels and at 1000 frames per second and then edited to focus on the relevant segments.

### Micro-rheological experiment

To examine the rheological properties of regolith simulant, we laser cut a series of two dimensional (2D) hourglass funnels^38^ from acrylic with different throat diameters *D* incrementally spaced from 0.75 to 1.75 mm in 0.25 mm steps (Fig. 2A). Each hourglass is 45 mm tall and split into three sections of length 15 mm. The top and bottom sections are square reservoirs with a width of 15 mm. These reservoirs connect to the central throat region through symmetric hoppers, each with an opening angle of *α* = 75^◦^ such that the throat is a rectangular channel 3.5 mm long. Each section is laser cut via a raster technique to a uniform depth of 0.75 mm. These micro hourglasses are filled with 0.5 mg of Mars wind drift regolith simulant, and their open faces are sealed with a glass microscope slide. The funnels are mounted on a stepper motor set to rotate by 180^◦^ every 6 s. This duration is a long enough interval such that every hourglass drains completely before the next flip. The particle flows through the hourglasses are imaged with a Phantom Miro C210 digital high-speed camera using a Nikon 60 mm Micro Nikor objective lens at 5.6× magnification resulting in a resolution of 5 µm per pixel (Fig. 2C, D). Videos are recorded at 500 fps, the particles are then tracked using ImageJ^67^ and the velocity flow profile of the draining regolith grains are measured via an in-house designed particle tracking system and recorded across the throat region for 4 consecutive flips (Fig. 2E, F).

To create flow profiles from these images, we detect the location of grains within the images. First, the images are cropped around the rectangular throat region of the hourglass, shown in Fig. 2D. Then, a binary threshold is applied to generate an image mask with the grains highlighted in white on a black background (Fig. 2E). We use a calibrated shape smoothing step to remove extraneous dots in the masks and smooth the edges of the detected grains^68^. Once these clean grain masks have been created, we use a trained function that finds contours^69^ to apply a bounding box around the images (Fig. 2F). The location and size of each bounding box is then saved to an image mask text file for each video frame.

The text files provide the location of every grain in every image. Therefore, with a known frame rate we can use that information to create flow profiles. We create a script to do this in Jupyter Notebook™ with Python™. First, we identify the same grains in consecutive images to extrapolate the distance the grain traveled between images. This was accomplished through a matching algorithm that compared the location of a bounding box with every bounding box in the previous frame and found the one that was closest. Additional matching parameters were added, such as a similar bounding box size and minimal movement in the y direction since the grains are known to fall straight down. This ensured that grain positions were accurately matched with their positions from the previous frame. We then calculate the distance each box traveled between images and multiplied by the frame rate to obtain the velocity of each. These velocities were then plotted as a function of x position across the throat.

### Numerical simulation

We construct a funnel composed of a conical section of height 40 cm with an upper opening 20 cm in diameter and a throat of diameter *D* varying between 0.75 mm and 1.75 mm and length 1 cm. Into this funnel we randomly pour 1500 spherical particles whose diameters are uniformly distributed between 0.5 and 0.005 mm^70^ (Fig. 4A). Each of these particles of radius ${R}_{i}$ has a mass of ${m}_{i}=\rho \frac{4}{3}\pi {R}_{i}^{3}$, where *ρ* = 400.0 kg*/*m^3^, the average density of the Martian simulant. The particles interact with each other and with the walls of the funnel only upon contact according to a damped hertzian-spring force of the form,

2 $${{\bf{F}}}_{{\rm{ij}}}=\left\{\begin{array}{cc}{{\bf{F}}}_{{\rm{ij}}}^{{\rm{n}}}{\boldsymbol{+}}{{\bf{F}}}_{{\rm{ij}}}^{{\rm{t}}} & {\delta }_{{ij}}\le 0\\ 0 & {\delta }_{{ij}} > 0\end{array}\right.$$

where

3 $${{\bf{F}}}_{{\rm{ij}}}^{{\rm{n}}}=\sqrt{{\delta }_{{ij}}}\sqrt{\frac{{R}_{i}{R}_{j}}{{R}_{i}+{R}_{j}}}\left({K}_{n}{{\boldsymbol{\delta }}}_{{ij}}-{m}_{{\rm{eff}}}{\gamma }_{n}{{\bf{v}}}_{{ij}}^{n}\right)-2\pi \phi \frac{{R}_{i}{R}_{j}}{{R}_{i}+{R}_{j}}$$

4 $${{\bf{F}}}_{{\rm{ij}}}^{{\rm{t}}}=-\min \left[\mu {{\bf{F}}}_{{\rm{ij}}}^{{\rm{n}}},\sqrt{{\delta }_{{ij}}}\sqrt{\frac{{R}_{i}{R}_{j}}{{R}_{i}+{R}_{j}}}\left({K}_{t}{{\mathbf{\Delta }}{\boldsymbol{s}}}_{{ij}}-{m}_{{\rm{eff}}}{\gamma }_{t}{{\bf{v}}}_{{ij}}^{t}\right)\right]$$

The degree of compression between two particles *i* and *j* at positions **r**_*i*_ and **r**_*j*_, and of radii *R*_*i*_ and *R*_*j*_, respectively; is quantified by the overlap ${{\boldsymbol{\delta }}}_{{ij}}={{\bf{r}}}_{{\rm{i}}}-{{\bf{r}}}_{{\rm{j}}}-\left({R}_{i}+{R}_{j}\right)$. The effective mass is defined as${m}_{{\rm{eff}}}=\frac{{m}_{i}{m}_{j}}{{m}_{i}+{m}_{j}}$, while ${{\bf{v}}}_{{ij}}^{n}$ and ${{\bf{v}}}_{{ij}}^{t}$ denote the relative normal and tangential velocities of the particles^71^ (Fig. 4B).

Focusing on the normal component of the interaction as shown in Eq. 3, its first term ${K}_{n}{{\boldsymbol{\delta }}}_{{ij}}$ produces an elastic spring-like repulsion which we match to the elastic response of lunar regolith via ${K}_{n}\,=\,4G/3(1\,-\,\nu ),$ where the shear modulus *G* is determined by ${G}={E}/2(1\,+\,\nu )$ for which we use *E* = 60 MPa for the Young’s modulus and $\nu \,=\,2/7$ for the Poisson ratio^58^. The second term, $-{m}_{{\rm{eff}}}{\gamma }_{n}{{\bf{v}}}_{{ij}}^{n}$, within Eq. 3 produces a normal damping force proportional to the normal velocity difference ${{\bf{v}}}_{{ij}}^{n}$ between the two particles controlled via the damping constant

${\gamma }_{n}\,=\sqrt{(\alpha 2{K}_{n})/m(1\,+\,0.25\alpha )}$, where $\alpha \,={\left(-2\frac{\mathrm{ln}\epsilon }{\pi }\right)}^{2}$ and *ϵ* is the coefficient of restitution of the particles, a parameter which we vary between 0.1 and 0.9. In this study, low values of *ϵ* correspond to very dissipative inter-particle interactions whereas high values correspond to very conservative interactions. The third and final term, $-2\pi \phi ({R}_{i}{R}_{j})/({R}_{i}+{R}_{j})$, produces an attractive potential in proportion to the effective radius between the two particles and scaled by a constant *ϕ*, the interaction energy of the two surfaces. This form approximates the Van der Waals interaction between two spheres at contact as originally calculated by Derjaguin^55^ and is the singular source of grain cohesion in our model as triboelectric or other electrostatic forces are not included^45^.

Moving onto the tangential component, Eq. 4, we see that it is the minimum of two terms. The first term of which, $\mu {{\bf{F}}}_{{ij}}^{n}$, is a Coulomb friction force governed by *µ*, the second parameter which we vary from 0.25 to 0.90. Here large values of *µ* imply strong inter-particle frictional forces impeding particles from sliding past each other, whereas low values imply particles can slide easily. The second term within Eq. 4 is the tangential version of ${{\bf{F}}}_{{ij}}^{n}$, where each normal term is replaced with its tangential variant; ${K}_{t}=\,4G/2(1\,-\,\nu )$, ${\mathbf{\Delta }}{{\boldsymbol{s}}}_{{ij}}$ is the tangential displacement between the two particles as truncated by the frictional yield criterion, ${\gamma }_{t}=\,{\gamma }_{n}/2$ is the tangential damping constant, and ${{\bf{v}}}_{{ij}}^{t}$ is the tangential velocity difference. Additionally, we apply a gravitational acceleration to each particle in the negative y direction of strength ${g}=\,[\mathrm{1,0.38,0.16,0.1,0.05,0.01}]{G}$, where G = 9.8 *m/s*^2^, corresponding to terrestrial, martian, lunar, and 3 sub-lunar gravitational accelerations. The simulation is made effectively two dimensional (2D) by only permitting grains to travel in x and y directions, and the funnel is initially sealed at the bottom end of the throat such that the particles may initially fill it up. To start the molecular dynamics simulation the seal is deleted and then the equations of motion of the particles are integrated through the velocity-Verlet algorithm within the simulation package LAMMPS using a time step of $250\pi \sqrt{2K_n/{m}-\,\gamma_n^2/4\,}=\,1.1359\,\times \,{10}^{-7}$ s for $22.5/{G}\times \,{10}^{6}$ time steps. Under some conditions the funnel jams and the particles do not move, under other conditions the particles flow out of the funnel in a continuous or quasi-continuous fashion.

## Supplementary information

Supplementary Information

Supplementary video1

Supplementary video2

Supplementary video3

Supplementary video4

Supplementary video5

Supplementary video6

## Data Availability

The data supporting the findings of this study is available from the authors upon reasonable request.
