# Supplementary material for: Behaviors of lunar regolith simulants under varying gravitational conditions
Source: NPJ Microgravity. 2025 Oct 1;11:69. doi: 10.1038/s41526-025-00501-z (PMC12489093; doi:10.1038/s41526-025-00501-z)
Supplement: Supplementary file 1 — Supplementary Information [file 41526_2025_501_MOESM1_ESM.pdf]

Supporting Information

for

Behaviors of Lunar Regolith Simulant Under Varying Gravitational Conditions

Ian P. Madden[1,2*], Sathyashri Muruganandam[2,3],

Amine Missaoui[1,2], Oliver Gries[4†], Jonathan Kollmer[4†],

Olfa D'Angelo[5,6†], Suman Sinha-Ray[1,2*]

List of Supporting videos:

- JSC1A_HougrlassFlowComparison_withText: Hourglass flow experiment on Earth and in lunar gravity provided by the ZARM GTB Pro. Material used is JSC 1A lunar simulant in an hourglass made from aluminum (side walls) and glass (front and back panel). The opening of the hourglass is 10mm wide, distance between front and back wall is also 10mm. Videos are captured at a resolution of 1024 × 768 pixels and at 1000 frames per second and then edited to focus on the relevant segments. Acknowledgement: DLR German Space Agency, BMWK Grant 50WM2342B.

- Simulation_EarthGravity_Phi0.001: Simulation of 2D flow for a cohesive material, with $\varepsilon = 0.95$, $\mu = 0.75$, and $\phi = 0.001$, at 1.0 G. 16 replicants of the same simulation are recorder over 3.10 minutes, the only difference between these replicants is their initial random packing of the granular material. A frame is taken every 5000 time-steps for a total of 4,500 frames per replicant. This material flows through the hopper much like the material flows through the micro-rheological experiment.

- Simulation_LunarGravity_Phi0.001: Simulation of 2D flow for a cohesive material, with $\varepsilon = 0.95$, $\mu = 0.75$, and $\phi = 0.001$, at 0.16 G. Similar recording procedure as previous movie. This material flows through the hopper in a very viscous, stopping and starting like manner indicating the cohesive force is starting to prevent flow.

- Simulation_OneTenthLunarGravity_Phi0.001: Simulation of 2D flow for a cohesive material, with $\varepsilon = 0.95$, $\mu = 0.75$, and $\phi = 0.001$, at 0.016 G. Similar recording procedure as previous movie. This material no longer flows through the hopper at all, it is clogged/jammed at these conditions.

- MicroExp_0.75mm: Micro-rheology experiment performed with Martian simulant using an opening diameter of 0.75mm, recorded at 500 frames per second, played back at 25 frames per second, with a resolution of 5 microns per pixel. Multiple flips occur during the movie however some are out of frame.

- MicroExp_175mm: Micro-rheology experiment performed with Martian simulant using an opening diameter of 1.75mm, recorded at 500 frames per second, played back at 25 frames per second, with a resolution of 5 microns per pixel. Flow duration is considerably shorter because the same amount of material is flowing through a much larger opening.

Supporting Materials:

- Determination of steady state flow in micro rheology experiment: The number of particles within the frame of the highspeed camera was counted per frame for the duration of one flip of a 0.75mm diameter hopper of duration 5s (Figure S1 A). During the middle 50% of this duration (1.25-3.75s, highlighted in yellow) the particle counts are semi-stable and so during this period we measure their velocities. After 3.75s a long non-zero tail exists due to straggler particles which we do not want to include in the flow velocity data. Similar middle 50% shown for a 1.75mm diameter hopper (Figure S1 B).

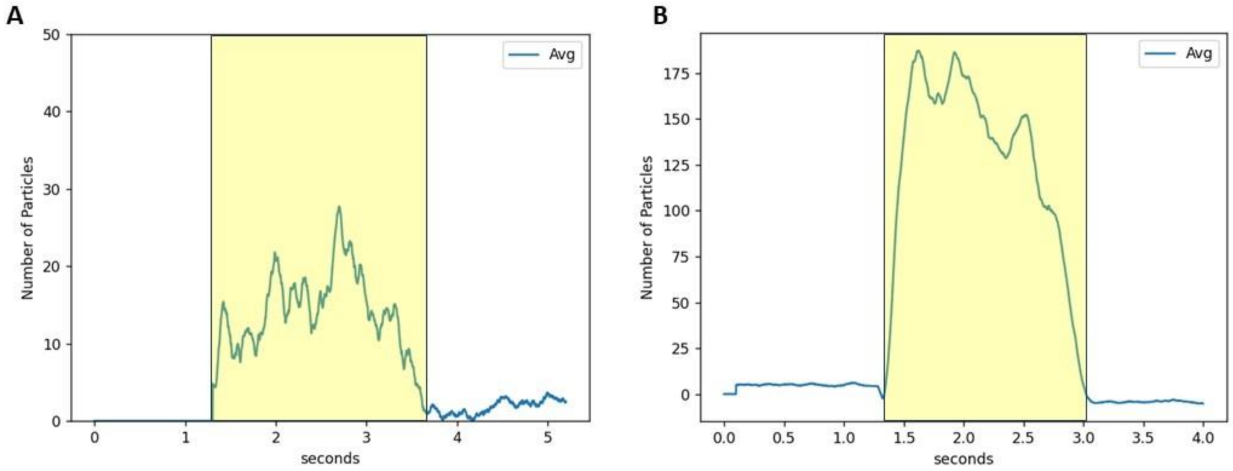

Figure S1: Particle count within smallest (A~0.75mm) and largest (B~1.75mm) hopper during a single flip. Yellow region corresponds to the middle 50% of the time data which was used to calculate flow velocities.

- Characteristics of the granular interparticle potential from simulation: For simulations performed under standard gravity conditions (1.0G) with $\varepsilon = 0.95$, $\mu = 0.75$, and $\phi = 0.001$ we recorded the distributions of average normalized intergranular compression throughout the duration of

the simulation calculated via $\frac{\delta_{ij}}{R_i+R_j}$, where δ_{ij} is the degree of overlap between two grains of nominal radius R_i and R_j (Figure S2, red data). We observed particles rarely overlap by more than 0.996% of their nominal radii indicating these inter-granular potentials do indeed reproduce the dynamics of "hard" aka non-compliant spheres. We also concurrently calculated the Tabor parameter $T = \left(\frac{16\left(\frac{1}{R_i}+\frac{1}{R_j}\right)^{-1}\phi^2}{9K^2\delta_{ij}}\right)^{\frac{1}{3}}$, where $K = \left(\frac{2}{3}\right)\left(\frac{E}{(1-\nu)^2}\right)$, during the simulation (Figure S2, blue data) and observed the values remain exceedingly small around 1.3e-5. This indicates that the DMT model is an accurate representation of the inter sphere potential between hard grains for this choice in parameters.

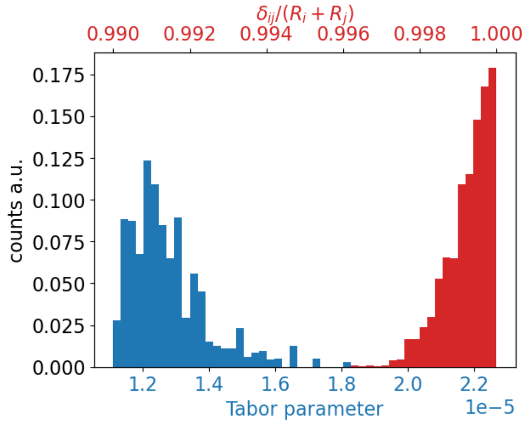

Figure S2: Distributions of Tabor parameters and particle overlaps for a simulation performed at $\varepsilon = 0.95$, $\mu = 0.75$, *and* $\phi = 0.001$. Particle overlap distribution $\frac{\delta_{ij}}{R_i+R_j}$ (red data) is highly skewed towards 1.0 showing particles are never compressed to more than 0.996 times the nominal radii, indicating a sufficiently hard particle. Similarly, the Tabor parameters measured on a per particle basis (blue data) are all significantly small, clustered around 1.3e-5.

We then repeated this analysis for a different set of parameters, $\varepsilon = 0.7$, $\mu = 0.5$, and $\phi = 0.1$, but found a similar series of distributions indicating the simulations is accurately representing the collisions between hard spheres (Figure S3).

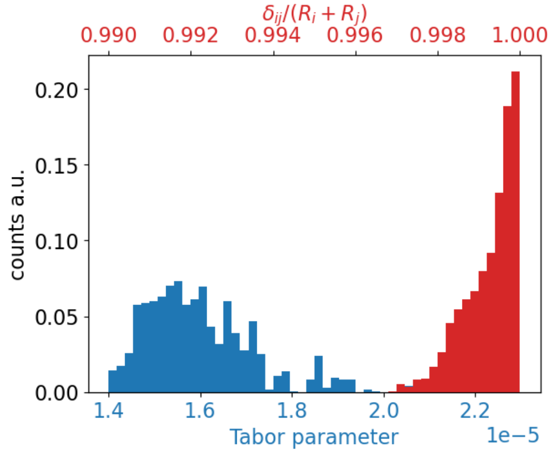

Figure S3: Distributions of Tabor parameters and particle overlaps for a simulation performed at $\varepsilon = 0.7$, $\mu = 0.5$, *and* $\phi = 0.1$. Particle overlap distribution $\frac{\delta_{ij}}{R_i+R_j}$ (red data) is again highly skewed towards 1.0 showing particles still are never compressed to more than 0.996 times the nominal. The Tabor parameters measured on a per particle basis (blue data) are also all significantly small, clustered around 1.5e-5.

- Influence of friction on particle dynamics: As seen in Figure 6 in the main text, friction has little impact on the observed flow profiles. We investigate this lack of influence further here by tracking the flow profiles over time for simulations performed at Lunar gravity but with varying friction coefficients (Figure S4). As expected, friction strongly effects the flow early on during the initial period of the simulation, however the variation dissipates at the simulation proceeds, we attribute this to the very small amount of regolith used.

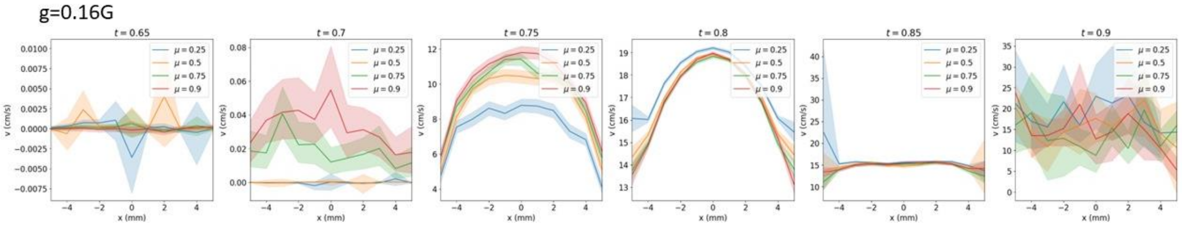

Figure S4: Velocity profiles measured at various times, 0.65%-0.9% duration of the full simulation, for simulations at Lunar gravity and for $\mu = 0.25 - 0.9$. Variations in flow are strongest during the initial and middle parts of the simulations, but then these variations die down as the funnel drains to completion.
